# Disturbances of the Gut Microbiota and Microbiota-Derived Metabolites in Inflammatory Bowel Disease

**DOI:** 10.3390/nu14235140

**Published:** 2022-12-02

**Authors:** Yongjia Hu, Zhouzhou Chen, Chengchen Xu, Shidong Kan, Daijie Chen

**Affiliations:** 1 School of Pharmacy, Shanghai Jiao Tong University, No. 800 Dongchuan Road, Minhang District, Shanghai 200240, China; 2 State Key Laboratory of Microbial Metabolism, Shanghai Jiao Tong University, No. 800 Dongchuan Road, Minhang District, Shanghai 200240, China

**Keywords:** inflammatory bowel disease, gut microbiota, dysbiosis, microbiota-derived metabolites, probiotic

## Abstract

Inflammatory bowel disease (IBD), comprising Crohn’s disease (CD) and ulcerative colitis (UC), is characterized as a chronic and recurrent inflammatory disease whose pathogenesis is still elusive. The gut microbiota exerts important and diverse effects on host physiology through maintaining immune balance and generating health-benefiting metabolites. Many studies have demonstrated that IBD is associated with disturbances in the composition and function of the gut microbiota. Both the abundance and diversity of gut microbiota are dramatically decreased in IBD patients. Furthermore, some particular classes of microbiota-derived metabolites, principally short-chain fatty acids, tryptophan, and its metabolites, and bile acids have also been implicated in the pathogenesis of IBD. In this review, we aim to define the disturbance of gut microbiota and the key classes of microbiota-derived metabolites in IBD pathogenesis. In addition, we also focus on scientific evidence on probiotics, not only on the molecular mechanisms underlying the beneficial effects of probiotics on IBD but also the challenges it faces in safe and appropriate application.

## 1. Introduction

Inflammatory bowel disease (IBD), generally including ulcerative colitis (UC) and Crohn’s disease (CD), is a chronic and relapsing intestinal inflammatory disease. There are differences in the predilection sites between UC and CD. UC usually involves the distal colon and rectum, invading the intestinal mucosa and submucosa, while CD, a discontinuous inflammation disease, can attack the entire digestive tract, and its lesions can penetrate all the intestinal layers [1]. IBD tends to occur in developed countries, and epidemiological surveys show that its incidence has gradually increased in recent years globally. It was reported that, from 1980 to 2013, the incidence of IBD in Denmark increased from 15.9 to 27.7 per 100,000 people [2]. Furthermore, the incidence of IBD in the Netherlands increased from 17.51 to 38.96 per 100,000 people from 1991 to 2010, with an average growth rate of 4.30% annually [3]. In recent years, the prevalence of IBD has increased in Asia, and it shows a gradient trend of increase from north to south in China [4]. Although the pathogenesis of IBD is still elusive, it is generally believed to be related to genetic susceptibility, environmental factors, immune dysfunction, and gut microbiota [5,6]. It was reported that a high risk of developing IBD was observed in the offspring of affected parents [7]. Potentially relevant environmental factors, such as antibiotic exposure, smoking, dietary fiber, saturated fats, and major life stressors, are associated with IBD incidence [8]. In addition, gut microbiota dysbiosis was demonstrated in IBD patients and consistently characterized by a reduction in microbiota diversity compared with healthy individuals [9]. The use of existing therapeutic drugs, including sulfasalazine, mesalazine, glucocorticoids, and biological drugs, is limited to some extent due to their modest effects, adverse reactions, and high prices [10]. Therefore, new treatment strategies and methods have been called for in recent years.

The human intestinal tract harbors an estimated three trillion bacterial members, and the ratio of gut microbiota to human cells is about 1:1. However, the genetic diversity of gut microbiota is 100 times higher than that of human cells [11]. Gut microbiota is widely involved in human metabolic activities and plays an important role in maintaining immune and physiological balance. It was reported that primary resistance to immune checkpoint inhibitors, such as PD-1 and PD-L1, could be attributed to abnormal gut microbiota composition, and gut microbiota restoration improved the immunotherapy response of PD-1 blockade by recruiting more CCR9^+^CXCR3^+^CD4^+^ T lymphocytes into tumor beds [12]. In addition, gut microbiota disturbances and resulting metabolic disorders contribute to depression and diabetes via acting on the endocannabinoid system and decreasing glucose tolerance and insulin resistance [13,14].

More and more studies have shown that gut microbiota disturbance is closely related to the occurrence and development of IBD. Abnormal gut microbiota compositions were identified in IBD patients compared with healthy individuals, particularly with respect to the abundance and diversity of microbiota [5]. A large number of studies have also shown that IBD patients are usually accompanied by gut microbiota disturbance, which is mainly characterized by a decrease in the abundance and diversity of gut microbiota, among which the Firmicutes flora is reduced and the flora of Bacteroidetes and Actinomyces are increased [6,15]. In addition, gut microbiota dysbiosis could potentially induce intestinal inflammation by upregulating some pathogenic bacteria species, such as *Enterobacteriaceae*, increasing the production of lipopolysaccharides (LPSs) and activating inflammatory signaling pathways [16]. Therefore, relieving gut microbiota dysbiosis shows the potential to reduce the production of intestinal LPSs and ameliorate intestinal inflammation [17,18]. Metabolites derived from gut microbiota that have been proved to exert a protective role in humans also change in the context of IBD. To be exact, these metabolites are beneficial for promoting the maturation of the intestinal barrier and immune homeostasis. Therefore, a reduction in the metabolites derived from the gut microbiota leads to nutritional deficiency of the intestinal epithelial cells and aberrant immune function, thus injuring intestinal barrier integrity and accelerating IBD progression [19]. As gut microbiota regulators, probiotics have a certain therapeutic effect on intestinal inflammation in IBD through restoring the composition of gut microbiota and its derived metabolites [20,21]. In addition, probiotics are famous for their high level of safety, which shows good potential for intestinal diseases, and are expected to provide more options for the treatment of IBD. 

Although the associations between gut microbiota and IBD have been increasingly elucidated, the precise role of dysbiosis is still obscure. Definitive cause-and-effect mechanistic relationships have suffered from many limitations, making it difficult to provide a definition. In this review, we summarize the relationship between gut microbiota dysbiosis and IBD and pay more attention to the role of gut microbiota and its metabolic products in the occurrence and development of IBD. Furthermore, the therapeutic effects of probiotics on IBD are also systematically summarized. We believe our article can make a significant contribution to the literature because we not only decipher the changed gut microbiota composition in IBD, but focus on how this alteration can promote IBD by discussing the changed microbiota-derived metabolites and signaling pathway activity caused by receptors, such as TGR5, FXR, and AhR. Importantly, we also review the underlying mechanism of probiotics in ameliorating IBD severity. In addition, challenges that probiotics face to be used more securely and widely are also discussed in our manuscript, thus providing novel references for the development of probiotics in the future.

## 2. Methods

A literature search was carried out using the Web of Science and PubMed databases to retrieve relevant studies on the relationship between gut microbiota and IBD from the past 20 years. The most relevant studies, such as randomized controlled trials (RCTs), animal studies, and reviews, were included for the purposes of this review. The following keywords were used to search for articles: IBD, animal studies, colitis, gut microbiota, intestinal flora, metabolites, SCFAs, tryptophan metabolites, bile acids, and probiotic.

## 3. Gut Microbiota and IBD

The human gut microbiota is a complex and diverse community of viruses, fungi, and gut bacteria that inhabit the intestinal tract and participate in food digestion and internal environment maintenance. The gut microbiota is usually referred to as the intestinal bacteria, constituting the major part with up to 1000 bacterial species. Furthermore, gut microbiota is mainly composed of Firmicutes, Bacteroidetes, Proteobacteria, and Actinomycetes, of which the first two are the most abundant. It is estimated that the number of microbiota in the intestinal contents of the colon reaches to 10^11^–10^12^ CFU per gram [22]. Gut microbiota plays important roles in regulating human immune function, inhibiting the invasion of pathogenic microorganisms, and participating in human metabolic activities. The interaction between commensal bacteria and the immune system makes human immunity become mature gradually, which also shapes the gut microbiota structure to a relatively stable status. Therefore, the imbalance of this interaction can contribute to the pathogenesis of many disorders, such as IBD, systemic autoimmune diseases, cardiometabolic diseases and cancer [23,24]. The composition of gut microbiota can be affected by many factors, such as diet, age, and environment. At birth, the abundance of human gut microbiota is very low, and at the age of 9–12 months, the gut microbiota forms a complex structure due to the participation of factors such as diet and environment [25]. This complex bacterial structure is important for humans and helps to resist the intestinal microbiota changes caused by diet and antibiotics, keeping the intestinal environment relatively stable [26]. 

With the widespread application of next-generation sequencing, the relationship between gut microbiota and IBD becomes clearer. The sequencing results of clinical samples have shown that the pathogenesis of IBD is highly correlated with the disturbance of gut microbiota, which demonstrates that the abundance and diversity of gut microbiota in IBD patients are significantly lower than those in healthy humans [27,28]. Although the causal relationship between gut microbiota and IBD has been further confirmed in germ-free mice, the exact mechanism by which gut microbiota disturbance induces IBD has not been fully elucidated [29,30]. Based on the existing research results, gut microbiota disturbance is supposed to promote the occurrence and development of IBD through various pathways. 

### 3.1. Gut Microbiota Dysbiosis in IBD 

Gut microbiota dysbiosis refers to changes in the composition and structure of the gut microbiota. Many studies have shown that gut microbiota disturbance induces and promotes the occurrence and development of IBD [31]. Compared with healthy people, patients with IBD usually showed a microbiota imbalance with decreased gut microbiota diversity, reduced abundance of Firmicutes, and increased abundances of Bacteroidetes and Actinobacteria [27]. Bacteria sequencing in inflammatory and non-inflammatory colon tissue from the same CD patient showed that the diversity of the flora in the inflammatory sites was significantly lower than that in normal tissue [32]. Furthermore, by comparing the results of flora sequencing in the inflammatory and non-inflammatory tissue of IBD patients with tissues in the corresponding sites of healthy human, Hirano et al. [33] found that flora diversity was decreased both in the inflammatory and non-inflammatory colon tissues of IBD patients. In addition, the bacterial species of *Cloacibacterium* and *Tissierellaceae* were more enriched in inflammatory tissues compared with non-inflammatory sites in IBD patients [33,34]. 

Numerous studies have shown that the abundances of specific bacteria in the guts of IBD patients were altered. For example, opportunistic pathogens, such as *Enterobacteriaceae*, are significantly increased in IBD patients and are positively correlated with the severity of IBD, which can increase the production of LPS and induce colon inflammation by stimulating the release of proinflammatory cytokines [16,35]. As an opportunistic pathogen, *Clostridium difficile* could induce intestinal infection, prolong hospital stays, and aggravate the symptoms of patients with IBD. In addition, IBD patients infected with *C. difficile* showed more significant gut microbiota dysbiosis, indicating an interaction between *C. difficile* and commensal bacteria [36]. Interestingly, *C. difficile* is a bacterium that causes infection of the large intestine, which often affects people who have been taking broad-spectrum antibiotics for a long time. It is supposed that microbiome dysbiosis is the triggering factor of *C. difficile* infection, the severity of which could be ameliorated by fecal microbiota transplantation [37]. This could account for the more significant dysbiosis in IBD patients infected with *C. difficile* to a certain extent. Furthermore, evidence have shown that the abundances of beneficial bacteria species, including *Clostridium* clusters IV and XIVa, *Faecalibacterium prausnitzii*, and *Eubacterium*, were significantly decreased in IBD patients [38,39,40]. A meta-analysis conducted by Prosberg et al. demonstrated that patients with active IBD had lower abundances of *Clostridium coccoides*, *Clostridium leptum*, *F*. *prausnitzii*, and *Bifidobacterium* compared to patients in remission, which suggests that dysbiosis may be involved in the activity of IBD [41]. Gut microbiota interventions, such as *Lactobacillus* and *Bifidobacterium* administration, have been reported to ameliorate intestinal inflammation via promoting the production of anti-inflammatory cytokines, inhibiting the level of oxidative stress, and increasing the content of short-chain fatty acids, which have been ascertained in both IBD patients and in animal models [42,43]. 

### 3.2. Gut Microbiota Dysbiosis Leads to Immune Disorders

Intestinal commensal bacteria form a microbial barrier in the outer layer of the intestinal mucosa and maintain the balance of the mucosal immunology. It is reported that Th17 and Treg cells mediate the release of pro- and anti-inflammatory cytokines in host respectively, and the balance between Th17 and Treg is important for maintaining normal intestinal immunity [44]. Studies have shown that gut microbiota plays an important role in maintaining the balance between Th17 and Treg cells. The dysbiosis of gut microbiota leads to the imbalance between Th17 and Treg cell proportions, which makes Th17 cells dominate, leading to the increased release of pro-inflammatory factors and inducing intestinal mucosal inflammatory response [45]. Evidence from Segmented filamentous bacteria (SFB) showed that SFB could induce CD4^+^ T cells to differentiate into Th17 cells through the regulation of innate lymphoid cells (ILCs) and dendritic cells (DCs), which promotes the development of intestinal inflammation such as IBD [46]. SFB colonization of germ-free mice activates the production of antimicrobial defenses, such as IgA and antimicrobial peptides and proinflammatory cytokines, resulting in inflammation in colon [47]. Another intestinal commensal microbiome influencing T-cell homeostasis is *Clostridium* cluster XIVa and *Bacteroides fragilis*, which promoted the differentiation of Treg cells by inducing the expression of Forkhead box protein P3 (Foxp3), thus increasing the secretion of IL-10 and exerting anti-inflammatory effects [48,49]. The abundance of protective bacteria species such as *Bifidobacterium* and *Lactobacilli* was reported to decreased in IBD patients, and these protective bacteria could increase the frequency of mucosal Treg cells to ameliorate IBD symptoms [50]. Although Th17 cells contribute to the incidence and progression of IBD, the therapies aiming to block IL17 secreted by Th17 are not always satisfying. A double-blind, randomized clinical trial that included 59 patients demonstrated that blockade of IL-17A was ineffective and complicated with higher rates of adverse events compared with placebo, indicating the therapy to IBD patients should take more factors into account [51]. What’s more, gut microbiota manipulation can restore the normal structure of gut microbiota and regulate the aberrant immunity, showing a wider range of action. Studies have shown that a mixed preparation composed of four probiotics including *Lactobacillus reuteri* and *Bifidobacterium longum* was able to regulate the gut microbiota of mice with colitis, promote the expression of IL-10, and improve the symptoms of intestinal inflammation [52]. 

### 3.3. Gut Microbiota Dysbiosis Leads to Impaired Intestinal Barrier Function

Impaired intestinal barrier function promotes the occurrence and development of IBD [53]. Symbiotic bacteria colonize the surface of the mucosal epithelial layer and form a microbial barrier, which can resist the invasion of pathogenic microorganisms and protect the health of the host through colonization resistance or modulation of the intestinal innate immune response [54]. Gut microbiota dysbiosis can lead to a relative increase in opportunistic pathogens and the impairment of intestinal microbial barrier function, increasing intestinal permeability and opportunistic pathogen invasion and, thereby, inducing inflammatory responses in the colon [55]. A related study showed that, compared with healthy mice, germ-free and antibiotic-treated mice had increased sensitivity to dextran sulfate sodium (DSS), which aggravated intestinal epithelial damage [56]. 

In addition, gut microbiota disturbance in IBD patients, such as a decrease in Bacteroidetes and increases in *Peptostreptococcaceae* and *Enterobacteriaceae*, led to a decreased content of intestinal antimicrobial peptides (AMPs), including REG3G and DEFB1, which weakened intestinal defense barrier function as a consequence [57]. One study showed that gut microbiota activated Th17, as well as DCs, to enhance mucosal barrier integrity and inhibit intestinal infection [58]. In addition, an impaired intestinal mucosal barrier increases the sensitivity of intestinal epithelial cells to opportunistic pathogens. Once stimulated by the flagellin and lipoproteins of pathogens, Toll-like receptor 5 (TLR5) in DCs was activated to enhance the expressions of IL-22 and IL-23, which induced the occurrence and development of gut inflammation [59].

Tight junction proteins (TJPs), a vital component of the intestinal barrier, play a crucial role in maintaining barrier integrity by strengthening the connections between cells, including adherent junction and gap junction proteins. It is widely reported that TJPs, such as ZO-1 and occludin, are significantly decreased in IBD patients [60]. Meanwhile, gut microbiota interventions are indicated as effective methods to protect TJPs. A meta-analysis based on 47 collected articles on IBD in animals showed that probiotics were effective for improving TJP expression in inflammatory contexts of IBD animal models, among which *L*. *reuteri* demonstrated the greatest effect on claudin and ZO-1 expressions [61]. Therefore, dysbiosis induces and accelerates IBD progression by injuring intestinal barrier integrity, and gut microbiota intervention could alleviate this condition. 

## 4. Gut-Microbiota-Derived Metabolites and IBD

Gut microbiota is widely involved in host metabolic activities, producing a variety of active metabolites that play important roles in maintaining a host’s intestinal barrier integrity and immune balance via providing nutrition to intestinal epithelial cells and activating various receptors directly or indirectly. For example, gut microbiota is widely involved in the metabolism of carbohydrates, as well as tryptophan and bile acids (BAs), to generate short-chain fatty acids (SCFAs), indole derivatives, and secondary bile acid. Furthermore, gut microbiota helps to promote the biosynthesis of vitamin B and vitamin K in a host, showing a protective effect for the host [62].

**Figure 1:**
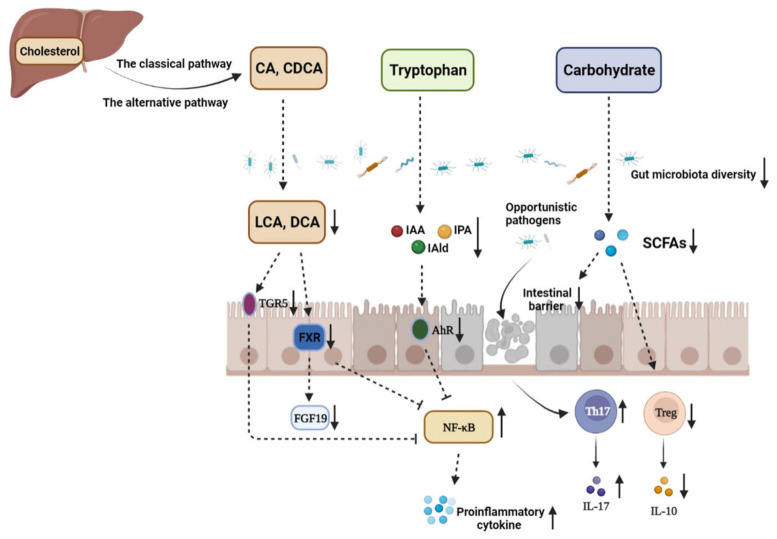
The disturbance of gut microbiota and microbiome metabolites in the pathogenesis of IBD. CA: cholic acid; CDCA: chenodeoxycholic acid; LCA: lithocholic acid; DCA: deoxycholic acid; TGR5: G-protein-coupled bile acid receptor 1; FXR: farnesoid X receptor; FGF19: fibroblast growth factor 19; IAA: indole-3-acetic acid; IAld: indole-3-aldehyde; IPA: indole-3-propionic acid; AhR: aryl hydrocarbon receptor; SCFAs: short-chain fatty acids. The decrease in the abundance and diversity of gut microbiota weakens the intestinal microbial barrier in IBD, which provides opportunistic pathogens an opportunity to invade gut mucosa and induce imbalance between Th17 and Treg cells, thus aggravating intestinal inflammation. The contents of metabolites, such as SCFAs, indole derivatives, and secondary BAs, derived from carbohydrates, tryptophan, and primary BAs under the action of the gut microbiota are significantly decreased due to gut microbiota dysbiosis. SCFAs mediate diverse effects on mucosal immunity, such as the maintenance of mucosal integrity, by supplying an energy source to colonocytes and expending Treg cell proportions. Therefore, SCFA reduction can aggravate intestinal injury in IBD patients. In addition, dysbiosis leads to loss of the microbial activation of tryptophan, which makes the endogenous ligands AhR, IAA, IPA, and IAld decreased. Gut microbiota disturbance in IBD can also decrease the contents of secondary BAs in the colon, such as LCA and DCA, which downregulates the activation of TGR5 and FXR and enhances NF-κB transcription, respectively.

In recent years, more and more studies have shown that the disturbance of the gut microbiota metabolic process is related to the occurrence and development of IBD. It is worth noticing that the contents of SCFAs in the intestines of IBD patients were significantly reduced compared with those of healthy people [63]. Studies have also found that the content of microbial-derived aryl hydrocarbon receptor (AhR) agonists is significantly reduced in IBD patients. In addition, supplementation with AhR agonists could significantly improve intestinal barrier integrity and ameliorate IBD symptoms [64]. There are increasing studies focusing on the role of gut microbiota in diseases, and alteration in the gut microbiota metabolic process is tightly associated with IBD (Figure 1). 

### 4.1. SCFAs and IBD 

The gut microbiota mainly depends on undigested food in the upper gastrointestinal tract to survive. Dietary carbohydrates are mainly fermented into SCFAs and gases by the gut microbiota. The three most common SCFAs in the gut are acetate, propionate, and butyrate. Among the SCFAs, butyrate is considered to be the most important component for human health as not only the main energy source of colonocytes, but also an important regulator of intestinal barrier integrity. In addition, butyrate promoted intestinal epithelial cell differentiation, tissue development, and immune balance [65]. Acetate is produced by many bacteria during food fermentation, while propionate and butyrate tend to be produced by specific bacteria. Firmicutes are the primary butyrate-producing bacteria, which include *Lachnospiraceae* and *F. prausnitzii* [62]. Evidence from 16S bacteria sequencing demonstrated that the abundances of *Lachnospiraceae* and *F. prausnitzii* were significantly reduced in the feces of IBD patients, resulting in decreased intestinal butyrate content and aggravated intestinal barrier damage in inflamed colons [66,67]. In addition, a relevant study showed that the abundance of *Roseburia hominis*, a butyrate-producing bacteria, was notably reduced in IBD patients, which was tightly related to the reduced content of SCFAs in the feces of patients [68]. A meta-analysis including eleven studies demonstrated that UC patients had dramatically lower total SCFA concentrations compared to healthy subjects, and the concentrations of acetate, propionate, and butyrate were different according to disease status. Active UC patients had reduced acetate and propionate concentrations, while UC patients in remission had similar concentrations to healthy subjects [69]. 

SCFAs are capable of promoting the differentiation and development of Treg cells by inducing Foxp3 expression or inducing DCs and intestinal epithelial cells to produce retinoic acids and TGF-β1, thus having a potential advantage in exerting anti-inflammatory effects in gut inflammation [70]. Similarly, in a randomized double-blind clinical trial, a topical application of SCFAs was proved to reduce endoscopic and histopathological scores and relieve inflammatory symptoms in UC patients [71]. TJPs play an important role in the maintenance of intestinal barrier integrity by strengthening the connection between intestinal epithelial cells and promoting the polarization of enterocytes. Many studies have shown that SCFAs, especially butyrate, can enhance the expressions of TJPs, such as Claudin-1 and ZO-1 in the colon, which are significantly decreased in the intestines of IBD patients [72]. As regulators of gut microbiota dysbiosis, probiotics and prebiotics are supposed to improve the diversity of gut microbiota and increase the relative abundance of SCFA-producing bacteria, resulting in alleviated severity of IBD [73,74].

### 4.2. Tryptophan Metabolism and IBD

**Figure 2:**
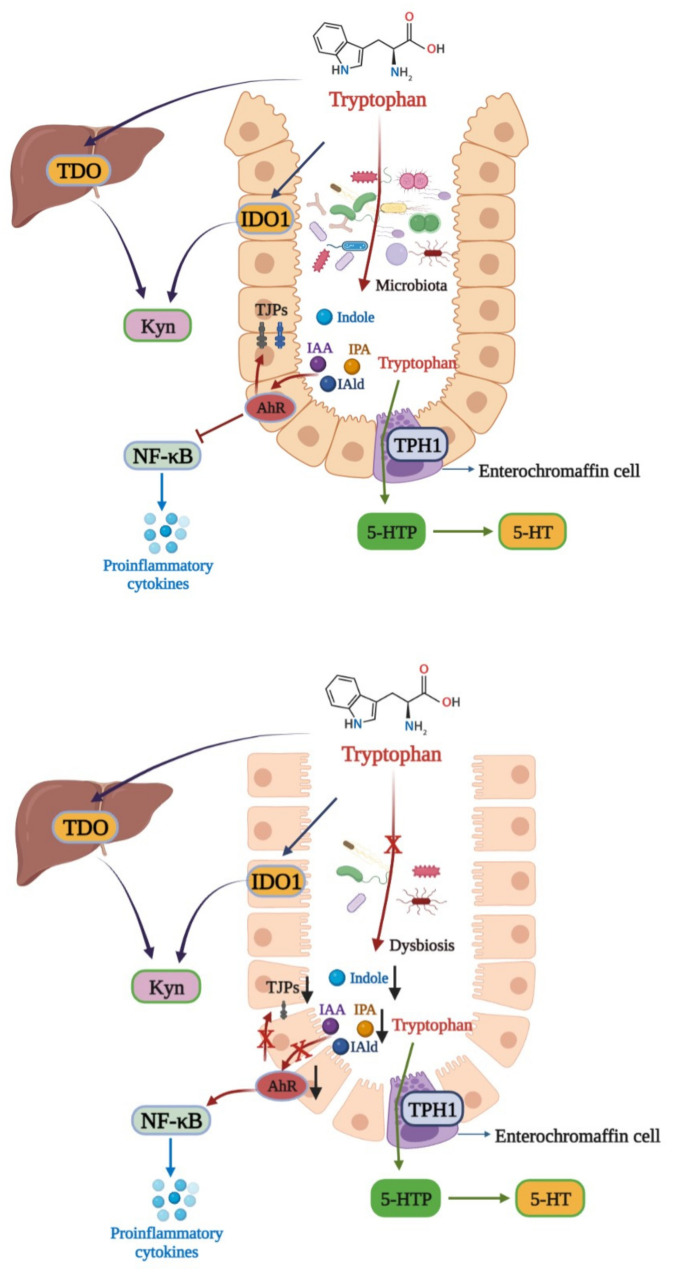
Tryptophan metabolism disturbance in IBD. There are three main metabolic pathways of tryptophan. The major pathway is the Kyn pathway, with tryptophan also being metabolized into indole derivatives. In healthy individuals, the gut microbiota metabolize tryptophan into IAA, IPA, and IAld, which can activate AhR to exert a protective effect in the colon via inhibiting NF-κB and increasing TJPs expressions. In IBD, gut microbiota dysbiosis leads to the microbial activation of tryptophan, aggravating intestinal inflammation. TPH1: tryptophan hydroxylase 1; 5-HTtp: 5-hydroxytryptophan.

Tryptophan is an essential aromatic amino acid for the human body, and a host mainly obtains tryptophan from external intake. Tryptophan plays an important role in a host as a synthetic precursor of various biologically active substances, such as serotonin (5-HT), kynurenine (Kyn), and indole derivatives [75]. There are three main metabolic pathways of tryptophan in the body [76]. The first one is the Kyn pathway (KP), through which tryptophan is metabolized into kynurenine (Kyn) by indoleamine 2,3-dioxygenase (IDO) and tryptophan 2,3-dioxygenase (TDO). In the second pathway, tryptophan is metabolized into 5-HT by the tryptophan hydroxylase 1 enzyme (TPH1) in enterochromaffin cells. The third is the microbial pathway, where tryptophan is converted into indole and its derivatives under the action of the gut microbiota (Figure 2). Indoles act as endogenous ligands for AhR and can activate AhR to exert a wide range of physiological effects [75]. Due to their wide distribution and complex mechanisms, AhR and its endogenous ligands have become a research hotspot in recent years.

AhR is a member of the PER-ARNT-SIM (PAS) superfamily of transcription factors, which sense changes in the cellular environment and regulate the physiological balance of the body. It was reported that AhR inhibited the expression of NF-κB in a manner dependent on suppressor of cytokine signaling 2 (SOCS2) after activation to exert anti-inflammatory activity [77]. In addition, AhR maintains the integrity of intestinal and skin barrier activation by increasing the expressions of intestinal TJPs, such as ZO-1 and occludin, or activating the AhR-Nrf2 pathway [78,79]. Tryptophan can be decomposed into indoles and their derivatives under the action of the gut microbiota, such as IAA, IPA, and IAld [62]. These indole derivatives are able to activate AhR to protect the intestinal barrier and reduce the expressions of intestinal proinflammatory cytokines. 

In recent years, more and more studies have shown that disorders of tryptophan metabolism are strongly related to IBD [80,81]. In a clinical study, it was found that plasma tryptophan levels were decreased in IBD patients, and plasma tryptophan concentrations were negatively correlated with IBD severity [82]. Furthermore, the contents of AhR ligands derived from gut microbiota metabolism were significantly reduced in IBD patients, indicating the importance of endogenous AhR ligands in intestinal inflammation [83]. A similar finding was also revealed in AhR-knockout mice. Nikolaus et al. demonstrated that AhR-knockout mice were more sensitive to DSS-induced colitis, and supplementation with AhR ligands could improve the symptoms of colitis in mice [84]. Microbial-derived AhR ligands, such as IAA, IPA, and IAld, could activate AhR to reduce intestinal inflammation via inhibiting NF-κB signaling pathway activation and TNF-α expression in the intestines of IBD mice [85]. In addition, AhR activation was reported to increase the expressions of TJPs and promote wound repair in the intestinal tracts of UC mice [86]. 

As mentioned above, the gut microbiota is the primary source of endogenous AhR ligands. Some AhR ligands producing commensal microbiomes, such as *Peptostreptococcus russellii*, *Lactobacillus*, and *Bifidobacteria*, have been proved to be decreased in IBD patients [75,87]. Furthermore, Natividad et al. found that the administration of a *Lactobacillus* strain with high tryptophan-metabolizing capabilities could improve impaired microbiota-derived AhR ligand signaling in a host [88]. In general, gut microbiota dysbiosis induces tryptophan metabolite alteration to aggravate IBD progression, which is mainly based on reduced indole derivatives and AhR activity. However, due to the complexity of the gut microbiota, the microbiomes participating in tryptophan metabolism still deserve identification in the future. 

### 4.3. Bile Acids and IBD

**Figure 3:**
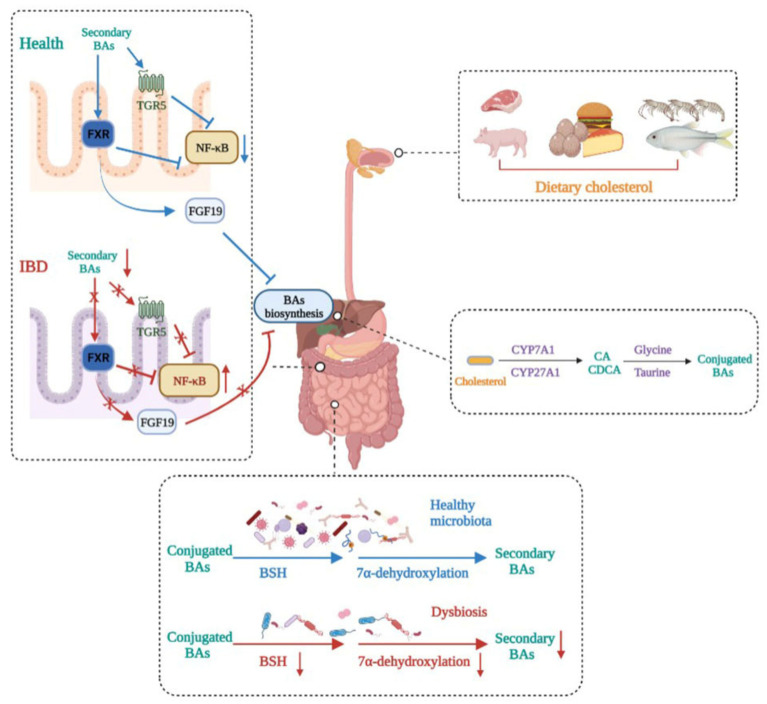
Bile acid metabolism disturbance in IBD. BA metabolism is altered in IBD patients. Dietary cholesterol is digested and absorbed in the gastrointestinal tract. Cholesterol is biotransformed to conjugated primary BAs in the liver and then secreted in the gut. Gut microbiota dysbiosis in IBD impairs BSH activity and 7α-dehydroxylation, leading to decreased secondary BAs. This induces decreases in the expressions of FXR and TGR5, which makes the transcription of NF-κB relevantly enhanced, thus aggravating IBD severity. In addition, decreased secondary BAs caused by dysbiosis reduce the production of FGF19, leading to primary BA accumulation in the liver.

BAs are synthesized from cholesterol in hepatocytes, and there are two main synthetic pathways, namely the classical pathway mediated by CYP7A1 and the alternative pathway mediated by CYP27A1, that generate primary BAs, such as CA and CDCA. Primary BAs are stored in the gallbladder and are subsequently secreted in the gut after conjugation to glycine or taurine. They can be transformed into secondary BAs under the action of the gut microbiota [89]. Secondary BAs, such as LCA and DCA, act as high-affinity ligands for TGR5 and FXR, the activation of which exerts immunomodulatory and anti-inflammatory effects [90]. Generally, there is a balance between primary BAs and secondary Bas, but the balance can be broken by gut microbiota dysbiosis, which causes increased primary BAs and decreased secondary BAs, inducing a state of inflammation in the colon (Figure 3).

A growing number of studies have shown that disturbances in bile acid metabolism are associated with IBD. Evidence from BA profiles found enhanced conjugated primary fecal BAs and decreased secondary fecal BAs in IBD patients, which could be caused by impaired deconjugation, transformation, or desulphation activities of the microbiota in IBD patients [91]. As high-affinity ligands for TGR5 and FXR, this BA alteration could lower the activation of TGR5 and FXR, which is detrimental to exerting an anti-inflammatory role. Meanwhile, the activation of FXR upregulated the expression of FGF19 in humans, a substance supposed to reach the liver and inhibit the synthesis of BAs, thus decreasing its toxicity effect on tissues [92]. Furthermore, it was reported that FXR and TGR5 activation showed anti-inflammatory effects by binding directly to an NF-κB p65 subunit to inhibit its transcription [89]. Macrophages, including M1 and M2 macrophages, are the main regulators of cytokine production in the gastrointestinal tract. TGR5 is a cell membrane receptor containing seven transmembrane domains, and the activation of TGR5 induces different reactions in M1 and M2 macrophages. The activation of TGR5 in M2 macrophages promoted immunosuppression by producing IL-10, leading to decreases in TNF-α and IFNγ [93]. However, TGR5 activation in M1 macrophages exerted the opposite effect and induced inflammation by strengthening NF-κB transcription and promoting proinflammatory cytokine production [89]. Despite the different roles of TGR5 in M1 and M2 macrophages, TGR5 activation exerts an anti-inflammation effect overall in macrophages.

The gut microbiota is widely involved in transforming BAs into unconjugated secondary Bas. Generally, conjugated Bas are converted into respective unconjugated free forms through the action of bile salt hydrolase (BSH)-carrying bacteria, such as *Bacteroides*, *Clostridium, Lactobacillus*, *Bifidobacterium*, and *Listeria*, the abundances of which have been demonstrated to decrease in IBD patients [50,89]. Subsequently, secondary BAs are formed from unconjugated BAs through the 7α-dehydroxylation of commensal bacteria, such as *Bacteroides*, *Clostridium*, *Eubacterium*, and *Lactobacillus* [81]. Therefore, gut microbiota dysbiosis occurring in IBD patients can cause BA metabolism alteration, mainly manifested as increased primary BAs and decreased secondary BAs, which aggravates IBD progression by inhibiting FXR and TGR5 activity. Therefore, the manipulation of BA balance shows great potential for IBD treatment. 

## 5. Probiotics and IBD

Probiotics are live microorganisms that have a beneficial effect on the body in sufficient doses. Probiotics are widely used in the adjuvant treatment of diseases because of their good efficacy and high safety. A large number of studies have shown that probiotics can improve the symptoms of both IBD patients and colitis animal models, showing great potential in the treatment of IBD. As gut microbiota regulators, probiotics were demonstrated to improve gut microbiota dysbiosis in IBD patients [6]. *Akkermansia muciniphila*, a new type of probiotic, was reported to increase the abundance of Firmicutes, decrease the level of gut inflammation, and restore gut microbiota structure in DSS-induced colitis mice [20]. Administration of VSL#3, a mixed probiotics product, ameliorated IBD significantly by maintaining tight junction protein expression and preventing apoptosis in a murine model of colitis [94]. As mentioned above, the balance between Th17 and Treg cells in the intestines of IBD patients is disrupted. *Lactobacillus plantarum* could stimulate the production of IL-10, increase the proportion of Treg cells, and exert an anti-inflammatory effect [95]. *F. praunsitzii*, a commensal butyrate-producing bacterium, had potential roles in gut homeostasis maintenance and in exerting anti-inflammatory effects on human IECs [96]. In addition, probiotic fermentation products also exhibit anti-inflammatory effects. Zhang et al. found that the severity of symptoms in DSS-induced colitis mice was reduced after the administration of milk fermented with *Bacillus subtilis*, the potential mechanism of which was that probiotic-fermented milk alleviated gut microbiota dysbiosis and increased the expressions of intestinal tight junction proteins in UC mice [73]. We developed a new kind of probiotic, selenium-enriched *B. longum* DD98, which was supposed to combine the properties of *B. longum* and proteinic selenium. The administration of Se-enriched *B. longum* DD98 significantly ameliorated colitis in mice through restoring gut microbiota structure, repairing intestinal barrier integrity, and inhibiting aberrant TLR4 activation in the colon, showing a better effect than both normal *B. longum* DD98 and sulfasalazine [97]. 

Probiotics can also exert a protective effect in IBD through increasing the contents of microbiota-derived metabolites. SCFAs, the energy source for intestinal epithelial cells, are able to protect the intestinal tissue and improve intestinal barrier function. Experiments showed that *Bifidobacterium* exerted anti-inflammatory effects in colitis mice by promoting the production of SCFAs [98]. A novel probiotic strain, *Lactococcus lactis* ML2018, was identified and isolated from traditional fermented food. *L. lactis* ML2018 was proved to inhibit DSS-induced intestinal inflammation in mice by improving intestinal barrier integrity, suppressing fibrosis, and upregulating the concentrations of SCFAs [99]. In addition, it has been shown that IBD patients are often accompanied by reduced AhR activity in vivo. *B. bifidum* administration played a positive role in increasing the contents of endogenous AhR ligands, which enhanced AhR activity to exert anti-inflammatory effects in DSS-induced colitis mice [100]. Similarly, Zhang et al. reported that *A. muciniphila* and its outer protein Amuc_1100 could significantly elevate the serum level of IAA and upregulate AhR-targeted genes in a DSS-induced mice model, suggesting that *A. muciniphila* and Amuc_1100 attenuated colonic inflammation by regulating tryptophan metabolism [101]. Probiotics can restore abnormal BA metabolism in IBD patients. The administration of Shirota strain of *Lactobacillus casei* could significantly ameliorate the severity of DSS-induced colitis by restoring the composition of the gut microbiota and circulating bile acid profiles in colitic mice [102]. 

Despite the good efficacy of probiotics in the treatment of IBD, their safe use still faces some challenges. *B. fragilis* is a novel probiotic that can colonize the intestinal mucosa and promote SCFA production to exert intestinal protective effects [103,104]. However, *B. fragilis* is also an opportunistic pathogen, among which enterotoxigenic *B. fragilis* poses a great threat to human health and is tightly associated with acute diarrhea, IBD, and colon cancer [105,106]. Furthermore, *B. fragilis* is resistant to penicillin by virtue of the production of beta-lactamase and other uncertain factors, increasing the risk of transforming resistance to humans [107]. Therefore, virulence factor detection and safety evaluations should be strengthened when developing *B. fragilis* probiotics. Montassier et al. found that, although the administration of probiotics could reduce antibiotic-resistant gene (ARG) numbers in the guts of colonization-permissive individuals, it exacerbated ARG expansion in the gastrointestinal mucosa, which were not encoded by probiotic strains [108]. Hence, further studies should be carried out to investigate the role of probiotics in the spread of ARGs along the human gastrointestinal tract. Altogether, although a large number of animal studies have indicated the protective effect of probiotics on hosts, there are still challenges for their safe use. New probiotics, such as *A. muciniphila* and *B. fragilis*, deserve more clinical data to support their efficacy and safety.

## 6. Conclusions

Efforts to date have effectively characterized the function of gut microbiota and microbiota-derived metabolites in healthy individuals, as well as those with IBD. However, the function of the gut microbiota, especially at the strain level, merits further studies. In this review, we summarized disturbance of the gut microbiota and microbiome metabolites in the pathogenesis of IBD. In addition, the roles of SCFAs, BAs, and tryptophan metabolites were subsequently discussed in detail. Moreover, microbiota-derived metabolites and their receptors in hosts that are possibly influenced by gut microbiota interventions, such as probiotics, represent promising targets for the development of novel therapeutic tools for IBD. With regard to future directions, more safety evaluations and clinical trials should be carried out in probiotics development to achieve these ambitious goals.
